# Barriers and Facilitators to Accessing Health Services for People Without Documentation Status in an Anti-Immigrant Era: A Socioecological Model

**DOI:** 10.1089/heq.2020.0138

**Published:** 2021-06-25

**Authors:** Riham M. Alwan, Dahlia A. Kaki, Renee Y. Hsia

**Affiliations:** ^1^ Division of Emergency Medicine, University of California, San Francisco, San Francisco, California, USA.; ^2^ School of Medicine, University of California, San Francisco, San Francisco, California, USA.

**Keywords:** health care access, undocumented immigrants, barriers to care, socioecological model, immigrant health, qualitative research

## Abstract

**Purpose:** This qualitative study explores the barriers and facilitators to health care from the perspective of providers who care for patients without documentation status in the San Francisco Bay Area.

**Methods:** Twenty-four direct providers were interviewed using semi-structured in-depth interviews. Participants included health care providers and community-based organization leaders. Interviews were independently coded using grounded theory analysis. The socioecological framework was used to develop the interview guide, analyze findings, and guide the discussion.

**Results:** Participants identified fear as a barrier that transcended multiple levels of influence. At the public policy level, national policies, such as public charge and anti-immigration rhetoric, limited access to services. Local expansion of health care coverage, such as Healthy San Francisco, facilitated access to care. At the organizational level, law enforcement presence generated fear. This was countered by a welcoming environment, described as culturally concordant clinical sites, representation of the community in the provider pool, and resources to address social needs. Individual-level fear, rooted in trauma and economic insecurity, was eased by trauma-informed care and health navigators. Community engagement and sustained partnerships built trust and credibility to transcend the fear that hindered access to care.

**Conclusion:** In a region with expansive policies for improved health care access, barriers are rooted in fear and span individual, organizational, and public policy levels of access to care. Richer community engagement may lessen the national and systemic barriers that this vulnerable population continues to face. Developing an understanding of this topic improves health care providers' ability to meet the needs of this growing and vulnerable population.

## Introduction

An estimated 10.5 million people without documentation status (PWDS) live in the United States, of whom 2 million reside in California.^1^ Access to health care for this population is limited. While numerous studies assess the impact of restricted health care access for PWDS, few account for the sociopolitical changes of the Trump administration and its impact in “sanctuary cities” such as the San Francisco Bay Area.^2^ This study uses qualitative methods to explore the barriers and facilitators to care from the perspective of providers who care for PWDS in the Bay Area to understand the effects of local policies on this population's health care access.

Health programs for PWDS are often underdeveloped and underfunded, resulting in significantly poorer health outcomes.^3^ Compared with the general population, PWDS have higher rates of hypertension, stress-related disease, and depression.^4–7^ This vulnerable population's lack of insurance, limited income sources, and “exposure to social environments with poor access to care, education, and health-promoting resources” are linked to these worse health outcomes.^3^ These challenges are manifested in concrete process measures, such as delays in the decision to seek care, difficulty in identifying and traveling to health care facilities, and receipt of lower quality care at those facilities.^8^

When the Affordable Care Act passed in 2010, 20 million people gained health care coverage, but PWDS were left out of the expansion, leaving cities, counties, and states to decide what health care provisions to extend to PWDS in their jurisdictions.^9^ As a result, policies and provisions vary widely.^10^ San Francisco, through the Healthy San Francisco comprehensive health care program, has been a leader in providing safety-net coverage plans (including primary and secondary medical care) to its residents regardless of documentation status or age, a feat matched by very few other municipalities.^11,12^

Nevertheless, barriers to care persist. Lack of insurance and other policy restrictions create an environment of fear and stress that dissuade patients from seeking care, even when absolutely necessary.^8,13–16^ The recent promulgation of the public charge rule, a federal regulation that limits the social services immigrants can utilize to qualify for legal permanent residency, has exacerbated this sense of fear and avoidance of the health care system.^2,17^ In addition to the policies that directly restrict health care access, several federal and local measures curtail social and economic opportunities for PWDS.^14^

These social determinants of health play a large role in determining access to care for PWDS. This includes fear of legal repercussions, language and cultural barriers, transportation and childcare costs, housing and education access, and so on.^8,18–20^ In some cities, community-based nonprofits focus interventions on the social determinants of health. Such organizations provide educational and social assistance (i.e., linkage to food, housing, and employment services) that ultimately improve health care access.^21^ Little data exist to highlight these facilitating services, however, making it difficult to assess success and scale up use of such programs.

This qualitative study explores and analyzes the barriers and facilitators to care from the perspective of the providers who care for PWDS in the Bay Area. Developing an understanding of this topic will improve health care providers' ability to provide effective emergent and non-emergent care that meets the needs of this vulnerable population.

## Methods

The institutional review board of the University of California, San Francisco determined all study procedures to be exempt. Ethical standards were upheld through the use of informed consent, confidentiality, and ability to withdraw at any point.

### Setting

Nine counties make up the Bay Area, a densely populated region of the United States and home to many immigrant communities. 23.5% of the region's population identify as Latinx.^22^ This region tends to vote Democratic and has some of the most liberal policies affecting health and employment of immigrants in the United States.^22^ This politically progressive region was chosen to explore the effects of progressive policies on PWDSs access to care.

### Participants

Participants were recruited via email, and initial participants were identified through the research team's professional contacts. Twenty-four employed adults with 3–40 years of experience were included in this study. Inclusion criteria were experience providing direct care for PWDS and affiliation with a community-based organization, school, or health center that served PWDS. Participants who did not speak English were excluded. Twenty-two participants currently work in the Bay Area. Three participants had lengthy experiences working in the Bay Area but now serve as consultants in nearby California regions. Snowball recruitment, where existing subjects recruit other participants, was utilized to increase the number of participants.^23^ No incentives were offered for participation.

### Data collection

The open-ended interview guide was developed and vetted by field experts and direct providers (Supplementary Appendix SA1). Semi-structured interviews, ranging from 45 to 60 min, were conducted and recorded on a password-protected virtual platform (Zoom) by investigators (R.M.A. and D.A.K.) between May and July 2020. Interviews were subsequently transcribed, and two researchers (R.M.A. and D.A.K.) double-checked the transcripts for accuracy and content.

### Data analysis

Grounded theory was used as the qualitative methodology to analyze the transcripts. A code is a label assigned to concepts found in the interviewee's narrative. Two members of the research team (R.M.A. and D.A.K.) independently reviewed and coded each interview upon completion of data collection, using Dedoose software. Through a series of meetings and transcription analyses, a codebook was built with agreement of the research team. Subsequently, investigators independently placed codes into broad categories. Through an iterative process and a series of weekly meetings, the group reached consensus on the categories and continued to independently code the interviews.^24–26^

Finally, the group convened for a collective compilation of categories into themes. While the socioecological framework was not used at the outset of the study, the model was found to best represent the relationship between themes in the data. Participant enrollment continued until saturation of themes occurred and no new information came forth, which is a standard procedure for estimating sample size for qualitative studies.^24^ A thorough process of member checking, which involves reviewing the data and the results with participants to check for accuracy and resonance with their experiences, was performed to ensure the credibility and trustworthiness of the findings. Measures were taken to promote credibility, transferability, and confirmability. Triangulation was utilized by reviewing the literature of the local region and observations of multiple cases of PWDS in the emergency room setting. The emergent themes of this study were reviewed by local community stakeholders, national experts.

## Results

A range of barriers and facilitators at multiple levels of the socioecological model of care likely influence PWDSs utilization of health care services. Health care providers in this study identified fear as an overarching barrier that transcends multiple levels of influence. Instigation of fear on a public policy level creates an atmosphere of lack of safety manifested on the organizational level. This void of safety for PWDS seeking health care leads to fear on the individual level. In this current political context, fear hinders PWDSs access to care.

### Public policy level of influence

At the highest level of our model, the most cited barriers to access to health care were government policies that limit access to services (Table 1).

**Table 1.** Public Policy Level of Influence

Descriptor	Quote
Fear due to national policies and anti-immigrant rhetoric	“There is a lot of fear and a lot of distrust because of just of all the policies that all of the government policies that have been in active for the last few years … there's a lot of mistrust and not knowing what services they can access … they often don't want to follow up because they don't want to sign up for insurance, or they think that those services that we provide will count against them. So, there's a lot of fear.” (Participant 5)
Fear due to public charge rule	“A lot has changed in the last 10 to 15 years around the anti-immigration sentiments that are coming through some of the political channels in government.” (Participant 10)
	“The whole point of it was really to instill fear … I don't think it's just the public charge. I think it's just the political climate in general.” (Participant 21)
Sanctuary city status as a facilitator to care	“I think San Francisco has bypassed a lot of potential national policies that might be anti-immigrant, and that may affect their health.” (Participant 3)
	“We're really lucky that we're in San Francisco and that there are policies regardless of your documentation status, if you're a resident, you do have access to a primary care doctor.” (Participant 2)
	“San Francisco has overtly stated repeatedly that they are a sanctuary city and that they are going to operate that way, that makes it easier for me at both of my sites to know that and to feel confident that … If I don't want to participate with ICE activities, that I don't have to … I find that quite empowering that I can protect my patients a little bit better.” (Participant 4)
Healthy San Francisco coverage as a facilitator to care	“I'm very happy with this program because they provide everyone's medical services, so when the clients don't have any insurance that they are able to go to Healthy San Francisco.” (Participant 19)
Restrictions on and limits to services	“I think this is one of the issues is that each county does things very differently. So, somebody in one county might have way better access than in another county.” (Participant 15)
	“It's not too hard to access care in the Pediatric Department, because in California, kids can get Medical. It's just regardless of your status. It's really amazing that we have that. I think for adults, it can be more challenging.” (Participant 21)

ICE, U.S. Immigration and Customs Enforcement.

Of note, the public charge rule was repeatedly described as a policy that instigated fear within communities of PWDS. Ramifications include decreased use of various social services (e.g., food stamps) in addition to a decline in use of medical care. One respondent reported:

“With all of the threats of the public charge, a lot of families, particularly adults, decided to disenroll from Medi-Cal for fear of being deported. So, that was also a big barrier to care, just sort of the political climate and what it does for people's trust in the medical system and accessing resources.” (Participant 21)

In this capacity, the Public Charge rule serves as a tangible consequence of the general xenophobic and anti-immigration rhetoric that has permeated national discourse throughout the Trump administration.

In contrast, a majority of respondents highlighted the advent of local measures aimed at counteracting and mediating the limitations imposed by national policy. Local policies such as the designation of San Francisco as a “sanctuary city” empowered providers to advocate for the safety of their patients in clinical spaces. Several participants lauded Healthy San Francisco, a county-level program geared to make affordable health services available to uninsured residents regardless of documentation status.

Nevertheless, such solutions were only reported at local levels, resulting in limits to services outside those geographic boundaries. Patients using Healthy San Francisco must be residents or employees of San Francisco County and can only receive medical services within city limits. Similar barriers arose between adult and pediatric populations. In California, all children younger than 25 years, regardless of documentation status, are eligible for Medi-Cal, which greatly improved access to care for pediatric populations. However, for their parents, or even as children “aged out” of the program, this presents a challenge.

### Organizational level of influence

Participants identified unsafe clinical spaces as a barrier for PWDS seeking care (Table 2).

**Table 2.** Organizational Level of Influence

Descriptor	Quote
Fear due to law enforcement in clinical spaces	“They're afraid to actually reach out to the resources because of all the ICE activities that have been happening or that were happening a few months ago … there's concern of going to a space that's more formal.” (Participant 3)
	“We've had people come back saying, ‘Well, I showed up to the place you sent me to, and there was a sheriff standing outside and he had a gun. And that did not feel like a place where I should be at this time, so I came back.’ … I sent them to our family health center, which they would very likely see a very kind, compassionate, culturally humble provider and staff, but that kind of symbol was enough to break that.” (Participant 2)
	“The organization has not committed either the resources or the time to teach doctors about trauma, and yet every clinic has a policeman.” (Participant 9)
Fear of being misunderstood in clinical spaces	“The biggest fear that we see mostly in immigrant communities is the fear of asking for help or the fear of even giving someone information when that person is trying to help them because in their mind, is that information going to go? How is it going to be used? Who's going to see it?” (Participant 24)
	“A clinic will develop a bunch of written materials in Spanish, and they're often at a very high reading level, and they don't recognize that a lot of undocumented immigrants don't have very good literacy. And so having somebody who can interpret our services that can be readily available is a key to that and could help develop that trust.” (Participant 10)
Welcoming clinical environment as a facilitator to care	“There's a big effort to try to make the clinic feel open and safe.” (Participant 3)
Language and cultural concordance as a facilitator to care	“When families come in, all of the front desk staff speak Spanish. It's a very sort of welcoming and friendly environment. So, I think a lot of families feel really comfortable and just that first touch point is key and critical to sort of help alleviate some of those fears.” (Participant 21)
	“It's one thing to say we serve folks and are willing to—It's another thing to make them feel like they're safe.” (Participant 20)
Hiring from within the community as a facilitator to care	“We hire explicitly from the community. So, we've had doctors whose parents were undocumented. So, really not just saying, ‘Hey, we open our doors to you.’ But actually building capacity with the community to serve themselves. That's been a motto that we really subscribe to.” (Participant 20)
Care coordination and social services as a facilitator to care	“Our HIV positive patient who comes undocumented … sending him to our legal partner and getting him that status and now he can work. That you change the course of people's lives when you're working together in coordination with others.” (Participant 20)
	“I'm just kind of coordinating referrals … I'm helping connect kids to services. So I'm figuring out, what's their Medi-Cal status? Who's their primary care provider? Do they need an appointment with an optometrist? Do they need an appointment with a dentist? Do they need to see a doctor? … And then also helping connect kids with lawyers in Alameda County to make sure they have a lawyer for their case … All of these processes are so involved … all these logistical things that when you're new to the country, it's just so complicated and confusing.” (Participant 22)
	“Asking families other things about them that aren't just their health but obviously impact their health … ‘Hey, do you have a lawyer? What's your food situation?’ All these other social determinants of health, once you address that in your clinic visit, it also helps sort of alleviate some of the other fears because it helps family see like, ‘Oh, you're actually seeing the whole picture or at least part of the picture.’” (Participant 21)

Fear of U.S. Immigration and Customs Enforcement raids and the presence of law enforcement in clinics contributed to a lack of safety within traditionally safe spaces, which subsequently strains the doctor–patient relationship. Patients seemed hesitant to share information that may be critical to managing acute and chronic illnesses. Compounded with the fear of deportation or detainment, PWDS also harbored a fear of being misunderstood in clinical spaces. A lack of adequate interpretation services and information contributed to this feeling.

All respondents reported organizational efforts to harbor a sense of safety in clinical spaces by creating a “welcoming environment” to moderate these organizational barriers. As described by providers, welcoming environments included three key features: cultural and linguistic concordance, care coordination, and social work services. A respondent explained:

“When they feel lack of fear inside, when they feel trust in their medical providers, when they see familiar faces. When they come in, they immediately feel like, ‘Okay, here I'm safe. My information is safe. People here look like me, speak my language, and they're going to help me without asking me all these questions about my status.’” (Participant 8)

Language and cultural concordant care were cited as key to addressing fear in clinical spaces. In particular, the presence of ancillary staff and providers, who represent the patients' community, alleviated patients' fears. Many providers noted that their organization's hiring practices sought providers who are underrepresented in medicine to create a pipeline of health care providers.

The presence of care coordination services in clinical spaces reportedly allowed PWDS a sense of safety and security in knowing their health needs will be addressed. The presence of social workers in clinical spaces eased access to care for PWDS and reassured patients that the clinical space is a place of safety and security. When social workers addressed food and housing insecurity, access to education, and offered partnerships to legal aid, PWDS felt safer and tended to return to such clinical spaces.

### Community level of influence

The barriers to care, particularly the barriers driven by fear, presented at all levels of the socioecological model were unanimously contrasted with community-level facilitators (Table 3).

**Table 3.** Community Level of Influence

Descriptor	Quote
Community-based partners as a facilitator to care	“I think the community-based organizations are huge. […] We have a lot of community-based organizations for almost every population that we have in San Francisco, even for very small populations. And those have been awesome.” (Participant 4)
	“We live in San Francisco and we have, we're very lucky in the sense that we have a lot of community based organizations that will do everything that they can to help patients navigate life, and obtain food resources, and help with finding employment.” (Participant 3)
	“Having the community linkages and having those readily accessible so that you don't need to reinvent the wheel for every patient, is extremely helpful. Understanding the school district and how it all works, so that you can help parents navigate that process is also very helpful. Wherever there are potential linkages to school nurses, counselors, also very helpful. Knowing which service providers in the community are going to be most appropriate and supportive of the families if you're making a referral for whatever service or mental health.” (Participant 6)
	“That's why the community clinics, where they're linked with the community, they have contacts in the community, they have familiarity and they're doing other kinds of events in the community. Those are the linkages I think that bring people in.” (Participant 14)
Community-based health navigation as a facilitator to care	“Oftentimes we find that the people who have the most credibility, who are most trustworthy, are people that come from within the community. I'm thinking about community promotoras. And the reason for that is because these individuals have lived experiences, but also they have a way of communicating with the community that we're trying to reach. […] One thing is to know that there's available services, but the other thing is how do you utilize them. And promotoras serve as the health navigators to do this.” (Participant 24)
Hiring from within the community as a facilitator to care	“I think that what's nice about [institution] is that it has been in the community for so long. A lot of the people who work there are from the community itself. So, a lot of the medical assistants, a lot of the front desk people, even some of the physicians as well. Because of that, the word spreads, based on who's hired there. I think that's a good learning point for clinics around the country is just if you want your clinic to have legitimacy where you are, it's good to hire people who are in your community and from your community, and that helps spread the word.” (Participant 21)
	“Hiring [community members] as staff and having their voices be a part of the clinic and then their faces also be representing the clinic. And that just changes the culture too. It allows the culture to more mimic your population that you're serving. They just become one in that way.” (Participant 15)

Building trust on the community level was reported as a challenging, multifaceted but effective approach:

“Trust is critical. When you build that trust, you have to sustain it, and you have to value it, because oftentimes, there's a lot of mistrust in these communities, especially with the way things are now with a lot of the political injustice that we're seeing. There's fear involved in [seeking care]. So with fear, trust …. is harder to achieve.” (Participant 24)

Almost all study participants cited the benefit of having community-based partners when providing care to PWDS. Respondents often listed a variety of community partnerships that they and their patients relied on, from legal services to mental health resources to cultural affinity groups. The relationship between providers and community-based organizations (CBOs) was described as a symbiotic one—providers relied on CBOs to fill gaps the health care system was not able to and CBOs, trusted resources in the community, referred patients to accessible and affordable care.

The health navigator model was repeatedly cited as a successful tool for improving health care access. Respondents recognized that, in this confusing health care system, patients benefited from having a guiding hand to help parse through various needs and concerns. For Latinx communities specifically, many respondents described the benefit of working with *promotoras*, or community members and leaders trained in health education.

The value of *promotora* programs underscored the importance of engaging communities in their health care systems. As described above (organizational level), a staff that represented the patient population, that spoke the language, and that understood the culture ultimately facilitated access to care. Respondents noted that this is best achieved when providers themselves are members of the community that is being served.

### Individual level of influence

Fear and health literacy were identified as the two main barriers to seeking care on the individual level of influence for the socioecological model of PWDSs access to care (Table 4).

**Table 4.** Individual Level of Influence

Descriptor	Quote
Fear due to concerns of deportation	“Fear and safety … if you're afraid that at any turn you might be stopped or picked up or separated from family then that's kind of first and foremost and will trump getting health or anything else.” (Participant 14)
	“I think that people absolutely are much less likely to present to the emergency department for care, they're less likely to present to primary care, establish primary care, less likely to sign up for other aid services for food and other kind of things if there is a fear of deportation.” (Participant 4)
Fear due to concerns of economic burden and loss of employment	“They may have employment that may not be flexible for them to be able to take time off work, to be able to seek healthcare … they don't have income levels that are going to provide them with extended options in terms of seeking healthcare, seeking other options, to make sure that they're safe and that they're healthy.” (Participant 18)
	“So fear essentially leads to patients not speaking up, not being advocates for themselves, not seeking care, and fear of also losing their jobs and economic stability. Another fear that I hear my patients share, are things like, ‘Well, I'm afraid that I'm going to be tracked after I come in contact with ‘the system.’” (Participant 16)
	“The fear of the economic burden is high. Every time that a bill comes, they have to make a choice. ‘Do I pay this bill, or do I save it for my rent, or do I buy food today?’ That's a very real economic fear. It's a big concern for people to access medical care.” (Participant 8)
Trauma-informed care as a facilitator to care	“The need for trauma-informed care in our clinicians is extraordinary and we have no training on it in our primary care, none.” (Participant 9)
Poor health literacy as a barrier to care	“I think a lot of that is access to information, providing the knowledge of what they need to know to navigate the system and advocate for themselves.” (Participant 18)
Health navigation programs as a facilitator to care	“Our family navigators will do a full social screening in multiple domains, including food, housing, work, transportation, and a couple of other domains. And then when they identify something, they kind of assess with the family like what are your priorities and where do you need help … how to go to an appointment or how to fill out a reduced bus fare application … The thing that's the most different for this model here at the clinic is the family navigators and being able to help them work through some of those social needs barriers and problems.” (Participant 11)

These fears led to delays in care and, at times, poorer health outcomes for PWDS. According to study respondents, individual fear is often rooted in immigrants' traumatic experiences, fear of deportation, and xenophobia. The economic burden of seeking care and fear of job loss if patients do not qualify for public aid programs also hindered interaction with the medical care system. One respondent described:

“A lot of immigrants internalize this [fear] and then they become fearful to get care until they're very, very sick.” (Participant 16)

Providers addressing physical and mental health with an approach that considered a patient's prior trauma were repeatedly cited as a way to mitigate fear on the individual level. Further training in trauma-informed care was recommended by multiple respondents.

Poor health literacy was also identified as a barrier to seeking care on the individual level. The process of insurance enrollment, physical navigation to appropriate care sites, and an understanding of preventative models of health care were cited as common challenges.

Health navigator programs, particularly those that utilized community health workers, promoted health literacy on the individual level, combating the barriers that are rooted in fear or misinformation.

## Discussion

In this qualitative study, we examined the barriers and facilitators to health care for PWDS from the perspective of providers in the Bay Area. This study was conducted in a region of the United States with particularly progressive immigration policies, often in direct opposition to those enacted by the Trump administration (2016–2020). Our findings show that barriers to care exist at three levels of the socioecological model to health care: (1) public policy, (2) organization, and (3) individual. Facilitators to care are present at these three levels, as well as the fourth level of the model: (4) community.

The primary barrier to care underscored in this study was fear. Public policies sow xenophobia and promote patients' fear of deportation. At the institutional level, a scarcity of vital resources, such as interpreters and linguistically appropriate medical literature, fuels patients' fear of being misunderstood or mistreated. At the individual level, difficult immigration histories, subsequent anxiety/depression, as well as socioeconomic instability cement fears of retraumatization as well as loss of employment.

Facilitators to care, at each level of the model, addressed these fears. For example, local sanctuary city policies mitigate restrictive federal immigration measures. Welcoming clinical environments, rooted in social justice and staffed with diverse providers, quell institutional sources of fear. Care coordinators, health navigators, and trauma-informed practices help alleviate fears arising at the individual level. The most critical source of facilitation to health care arose at the community level of the model. Providers enthusiastically cited the benefit of community partnerships, clinics embedded within the community, and community member empowerment as the best way to alleviate all levels of fears within the undocumented immigrant community.

Our study thereby indicates that the most critical intervention for improving access to health care for this vulnerable population is addressing and alleviating the fear that permeates all aspects of the health care experience. Fear at all levels prevents patients from seeking timely and quality care and therefore reduces the benefit of other social services associated with the health care experience. Until the United States' executive and legislative branches can address this fear barrier, the numerous resources offered to patients via health care systems will not be sufficient. Linkages to food security, employment opportunities, and housing resources that are often centered around health care and social services depend on alleviating this overarching fear.

Assessing the issue of access to health care for PWDS impacts this population and the general American public. Lack of preventative care, resulting in late-stage preventable disease, is an expensive taxpayer burden.^27–29^ PWDS also represent a significant portion of the labor workforce, ensuring their health also ensures the health of the American economy.^30^ Finally, and most critically, improving health care access for PWDS aligns with the Center for Disease Control's goal to “eliminate health disparities and improve the health of all groups.”^31^

These findings should be understood within the context of certain limitations. First, this study's findings are limited to the Bay Area and are intended to be transferable rather than generalizable. Despite this region's progressive culture, barriers *continue* to exist for PWDS and are possibly *more* restrictive in other parts of the United States. Furthermore, these findings are specific to a political administration that intentionally sowed xenophobic attitudes toward immigrant populations. Nevertheless, while future government administrations may contribute to improving the experience of PWDS (e.g., the Biden administration has discontinued the revised public charge rule), it is unlikely to rapidly address the deep-seated mistrust and barriers to health care systems that these communities experience. Like other qualitative research methods, interviews are limited by their nonrandom sampling of participants. Due to the nature of our respondent pool and the Bay Area demographics, this study primarily assessed the experiences of the Latinx community. While overlap may exist between the experiences of various immigrant groups, results may not be transferable to other communities. Finally, we focused our interviews on providers and while many had personal experiences with lack of documentation, this study lacked the voice of patients themselves. Future research in this domain should explore the patient experience.

## Conclusions

Despite the Bay Area's progressive health care policies and welcoming culture, barriers for PWDS continue to be rooted in fear and span individual, organizational, and public policy levels of access to care. A feeling of safety is paramount for patients to seek care, to navigate a new and foreign system, and to manage their and their families' medical and social needs. Transcending this fear hinges on building trust at all levels of the socioecological model, with an emphasis on solidifying and amplifying the work of community leaders and organizers.

## Supplementary Material

Supplemental data

## Supplementary Material

Supplementary Appendix SA1

## Abbreviations Used

CBO: community-based organization
ICE: U.S. Immigration and Customs Enforcement
PWDS: people without documentation status
