# Supplementary material for: Barriers and Facilitators to Accessing Health Services for People Without Documentation Status in an Anti-Immigrant Era: A Socioecological Model
Source: Health Equity. 2021 Jun 25;5(1):448–56. doi: 10.1089/heq.2020.0138 (PMC8252901; doi:10.1089/heq.2020.0138)
Supplement: Supplemental data [file Supp_AppendixSA1.docx]

**Research Aims**

1. To assess local health policy variations that undocumented migrants in Spain, Italy, and the U.S. experience while accessing healthcare
2. To explore the barriers and facilitators to healthcare access that undocumented migrants in Spain, Italy, and the U.S. experience from the providers’ perspectives
3. To explore the depth and breadth of emergent and non-emergent care provided to undocumented migrants from the providers’ perspective

**Interview Guide**

Could you describe your practice setting and how many years have you been working in this field?

What is your role in providing care for patients without undocumented status?

How do you know if a patient does not have documentation status?

Tell me about the differences you face in providing care for patients with vs. without documentation status.

What are things that make it easier for you to provide care for this population and what are things that make it more challenging?

Describe the challenges you feel people without documentation status may experience in accessing healthcare, both generally in San Francisco and in your practice setting specifically?

What do you think helps patients without documentation status access the healthcare system?

What tools or resources do you utilize to provide care for patients without documentation status?

What tools or resources do you wish you had access to in providing care for patients without documentation status? In an ideal world, what would the system look like so as to ensure ideal care for patients without documentation status?

How have all these considerations changed given the current COVID-19 pandemic?

We are interested in learning about provider resilience. How do you take care of yourself, both in your role as a provider for a vulnerable population and in the heightened context of this pandemic?

What is your motivation in caring for this population?

What sustains you in doing this work?
